# Physical Plasticity of the Brain and Deep Brain Stimulation Lead: Evolution in the First Post-operative Week

**DOI:** 10.3389/fsurg.2020.00055

**Published:** 2020-08-25

**Authors:** Anthony Martino, Olivier Darbin, Kelsey Templeton, Daniel Dees, Markus Lammle, Tatiana Torres, Dakota Williams, Dean Naritoku

**Affiliations:** ^1^Department of Neurosurgery, College of Medicine, University of South Alabama, Mobile, AL, United States; ^2^Department of Neurology, College of Medicine, University of South Alabama, Mobile, AL, United States; ^3^Department of Radiology, Tulane University, New Orleans, LA, United States

**Keywords:** neurosurgery, neuromodulation, electrode, movement disorders, pneumocephalus

## Abstract

**Background:** Deep brain stimulation (DBS) is a therapy for movement disorders and psychiatric conditions. In the peri-operative period, brain shift occurs as the consequence of events related to the brain surgery which results in post-operative lead deformation.

**Objective:** To quantify post-operative 3-dimensional DBS lead deformation after implantation.

**Methods:** In 13 patients who had DBS lead implantation, we performed preoperative magnetic resonance imaging (MRI), preoperative computed tomography (CT) scans after placement of fiducial markers, and post-operative CT scans immediately, 24–48 h, and 7 days after implantation. The MRI scans were used to define brain orientation and merged with CT scans. Lead deviation was determined relative to a theoretical linear lead path defined by the skull entry and target lead tip points.

**Results:** In the sagittal plane, we distinguished an initial period after surgery (<48 h) characterized by a deviation of the lead toward the rostral direction and a late period (over 1 week) characterized by a lead deviation toward the caudal direction. In the coronal plane, there was higher probability of lead deviation in the lateral than medial direction. During 7 days after implantation, there was net movement of the center of the lead anteriorly, and the half of the lead close to the entry point moved medially. These deviations appeared normative since all patients included in this study had benefits from DBS therapy with total power of charged comparable to those described in literature.

**Conclusion:** DBS lead deviation occurs during 7 days after implantation. The range of deviation described in this study was not associated to adverse clinical effects and may be considered normative. Future multicenter studies would be helpful to define guide lines on DBS lead deformation and its contribution to clinical outcome.

## Introduction

Deep brain stimulation (DBS) is an effective treatment for movement disorders (1) and some psychiatric conditions (2, 3). In DBS, an electrode lead is implanted in a selected area of the brain and a small amount of electricity is delivered locally. Although there is no uniformly accepted theory about the mechanism of action of DBS, the electricity applied by a pulse train modulates neuronal activity locally and distally, altering pathologic neuronal activity associated with the condition (4, 5). Normal neuronal activity also may be altered, causing adverse events.

Identification of the target location for the DBS lead is based on image-guided neurosurgery using preoperative magnetic resonance imaging (MRI) and computed tomography (CT) scans (6). The confirmation of implanted lead location is based on post-operative CT images merged with preoperative MRI images (7). Precision DBS lead positioning is important to optimize the benefits and minimize adverse events (8, 9). However, image-guided neurosurgical systems (INGs) precision is optimal at the conditions that the geometries of the skull and brain to be constant between the pre-operative and post-operative assessment (10).

During DBS lead insertion, a pneumocephalus is formed by the entry of air and accumulation of fluid between the dural membrane and cortices, causing a shift of brain tissue (11, 12). The subdural gap and brain plasticity can result in curvature of the lead. The change in curvature of the electrode can be evaluated by comparing immediate post-operative and CT scans taken weeks after the procedure (11, 13). We previously implemented a semiautomatic algorithm to quantify post-operative lead deformation based on brain imaging (13). As the DBS leads are flexible, tissue movement along the lead can be documented from the effect of tissue movement on lead shape. The amount of curvature of the DBS lead compared with the initial straight penetration can be used to quantify physical brain plasticity along the DBS lead (14). However, limited information is available on the normative values of the DBS lead deformation and during the first week after the procedure.

In this study, we investigated the DBS lead deformation during the first week after lead implantation to evaluate post-operative changes in physical brain plasticity (15) in a population of patient who benefited from DBS therapy with nominal total power of charge. Lead deformation was measured in 3 dimensions over time.

## Materials and Methods

### Patients

The study was reviewed and approved by the University of South Alabama Institutional Review Board. Over the duration of the study, data collection was completed in 13 patients (see Result section for population description) resulting in 15 lead tracks analyzed. Patients were enrolled were candidates for Deep Brain Stimulation therapeutic either to alleviate the symptoms for Parkinson disease, essential tremor or dystonia. Description of the patient population is given in the Result section.

### Study Overview

On the first day, each patient had a planning MRI scan, prior the implantation of fiducial markers, and a CT scan with the fiducial markers in place. The DBS lead implantation was planned and performed on the second day. After DBS lead implantation, the patient was transported from the operating room and an immediate post-operative CT (CT1) scan was made. Additional CT scans were made before hospital discharge at 24–48 h after lead implantation (CT2), and 7 days (CT3) after lead implantation when the patient returned for follow-up and implantation of the DBS generator.

The MRI and CT scans were analyzed in MATLAB, MathWorks, Natick, MA. The MRI scan was oriented in the sagittal, coronal, and transverse planes by referencing the anterior commissure, posterior commissure, and aqueduct. Each post-operative CT scan was merged on the MRI scan and underwent semiautomatic analyses to identify the DBS lead entry and target points of the DBS lead for subsequent analysis of the lead deviation from a virtual straight line between these 2 points and to calculate the volume of pneumocephalus.

### Magnetic Resonance Imaging

The 3-Tesla MRI scan (Ingenia v5.3, Philips, Franklin, TX) was performed from the vertex to hard palate to provide a 3-dimensional imaging data set for imaging-guided stereotactic intraoperative surgical navigation. The MRI scan protocol included axial 3-dimensional T2-weighted acquisition (3D TSE multishot; TSE factor, 124; matrix, 256 × 256 mm; voxel size, 1 mm; TR, 2,500 ms; TE, shortest; NSA, 2; flip angle, 90 degrees) and axial 3-dimensional T1-weighted acquisition (3D FFE multishot; TFE factor, 114; matrix, 250 × 250 mm; voxel size, 1 mm; TR, TE, shortest; NSA, 2; flip angle, 8 degrees).

### Computed Tomography

The 3-dimensional CT scans were performed from the vertex to hard palate using a 64-slice scanner (Brilliance 64, Philips). The helical CT scan protocol included a lateral scout view (pitch, 0.673 mm; collimation, 64 × 0.625 mm; rotation time, 0.75 s; matrix, 512 × 512 pixels). Axial image reconstruction was performed with a brain smooth UA filter (slice thickness, 3 mm; increments, 3 mm).

### Surgery

The DBS electrode lead placement surgery was performed with stereotactic guidance (Nexframe, Medtronic, Fridley, MN). After preoperative planning, bone fiducial markers were registered, the planned incision was marked, and surgery was performed with sterile technique, mild sedation, and local anesthesia. A burr hole was made with meticulous hemostasis. The dura was opened and tissue glue was applied to minimize cerebrospinal fluid loss. A burr hole cover (Stimloc, Medtronic) was secured to the skull for mini frame attachment, and the frame was navigated (StealthStation S7 System, Medtronic) and locked into position. The motor drive was attached and microelectrode recording needle was inserted to an initial position 10 mm above target. After mapping was completed, the lead (Lead 3387S-40, Medtronic) was placed and secured to the skull with a locking cap.

### Magnetic Resonance and Computed Tomography Merging

The MRI and CT images were merged to decrease the variation in brain orientation between the CT scans and ensure correct measurement of the lead location and pneumocephalus in the sagittal, coronal, and transverse planes. The MRI was used to define the initial orientation of the brain and was used to orient the CT scans. The anterior commissure, posterior commissure, and aqueduct were used create a transformation matrix for brain alignment in the sagittal, coronal and transverse planes. The aligned MRI was defined as the fixed vortex, and each CT scan was defined as a mobile vortex. We used software functions (Image Processing Toolbox, MATLAB) dedicated to registering multimodal 3-dimensional medical images to merge the CT scans to the fixed vortex of the aligned MRI. The original pixel size and slide thickness were preserved for the merged CT scans.

### Lead Position in the Skull

The axial CT scans were used to determine the electrode lead position in the. The skull entry point was identified on the axial plane that best met the criteria for inclusion of the lead on the density line from the skull border. The lead tip was identified on the last axial plane with nominal density before fading.

Analysis of axial images was started at the skull entry point coordinate E(x, y, z). A method that was based on region growing and nearest neighbor principles was used to extract and isolate lead and tissue areas of CT brain images (16). The region was grown iteratively from E(x, y, z) by comparing all unallocated neighboring pixels in the plane that was axial to the region. The difference between pixel vs. mean region intensity was used as a measure of similarity. The pixel with the smallest difference measured was allocated to the respective region. The iterative process was stopped when the difference between mean region vs. new pixel intensity became larger than a predefined threshold; for a Digital Imaging and Communications in Medicine (DICOM) file with standardized scales between 0 and 1, a threshold value of 0.1 enabled lead delimitation for all CT scans.

The center of mass of the lead was calculated and used to localize the lead in each plane. The center of mass coordinates were used in a similar calculation in the adjacent plane toward the lead tip; this was feasible because the lead trajectory was curvilinear, without any bifurcation or deformation that would exceed the lead radius section between 2 axial planes. The series of lead center of mass values that were determined between the skull entry site and lead tip defined the observed lead path (13).

### Lead Deviation From Linear Path

The lead skull entry site and target lead tip points were used to define a theoretical linear lead path. A line algorithm was used to determine the points of the 3-dimensional raster (scaled on the voxel dimension) that best approximated a straight line between the lead skull entry site and tip points (17). The maximum Euclidean distance between the observed and theoretical linear paths was used as a measurement of the curvature of the lead in the brain (13).

A circular histogram was used as graphic tool to provide a succinct view of the distribution of electrode lead curvature in distance and direction. Using a polar coordinate system, the frequency of bend over the dorsoventral axis was plotted against direction, and color bands were used to represent distance ranges. The direction of the longest spoke showed the bend direction that had the greatest frequency (13).

### Post-operative Pneumocephalus

The pneumocephalus formed during the DBS lead placement procedure was quantified using a semiassisted algorithm that was similar to the algorithm used to evaluate lead position, but the pneumocephalus had a less predictable shape between planes because of the presence of independent pockets and bifurcations. Therefore, a user-defined plane was selected to identify the initial pixel for each pneumocephalus and slice (13). Calculations of pneumocephalus volume were performed with statistical software (MATLAB R2013b, MathWorks, Natick, MA, USA).

### Outcome Measurement and Electric Charge From Stimulation

As there was no available scale to quantify the benefit and adverse events of DBS therapy between different clinical conditions, we defined the total power resulting from stimulation as an outcome measure. The power parameter indicative of therapeutic efficacy was calculated according to:

$$\begin{array}{l} \text{P}=\left(\frac{{\text{Voltage}}^{2}}{\text{Impedance}}\right)\times \left(\text{Pulse Width}\right)\times \left(\text{Frequency}\right), \end{array}$$

where P was total therapeutic power (18).

### Statistics

Data were reported as median and 25th and 75th percentiles. The Friedman non-parametric paired statistic was used to test whether individual morphometric features were dependent of the directionality and whether they were changed over time during the post-operative week of monitoring. Wilcoxon signed rank non-parametric paired statistic was used to test whether individual morphometric features were changed between 2 times. The Mann-Whitney non-parametric non-paired statistic was used to compare the changes in magnitude between morphologic features. The Spearman's correlation test was used to investigate the correlations between variables. Statistical significance was defined by *p* < 0.05.

## Results

### Population Description

Data set represented 15 DBS leads implantations in 13 patients. Population of patients included 5 patients with Parkinson Disease, 7 patients with essential tremor and 1 patient with dystonia.

### Lead Deviation

**Figure 1:**
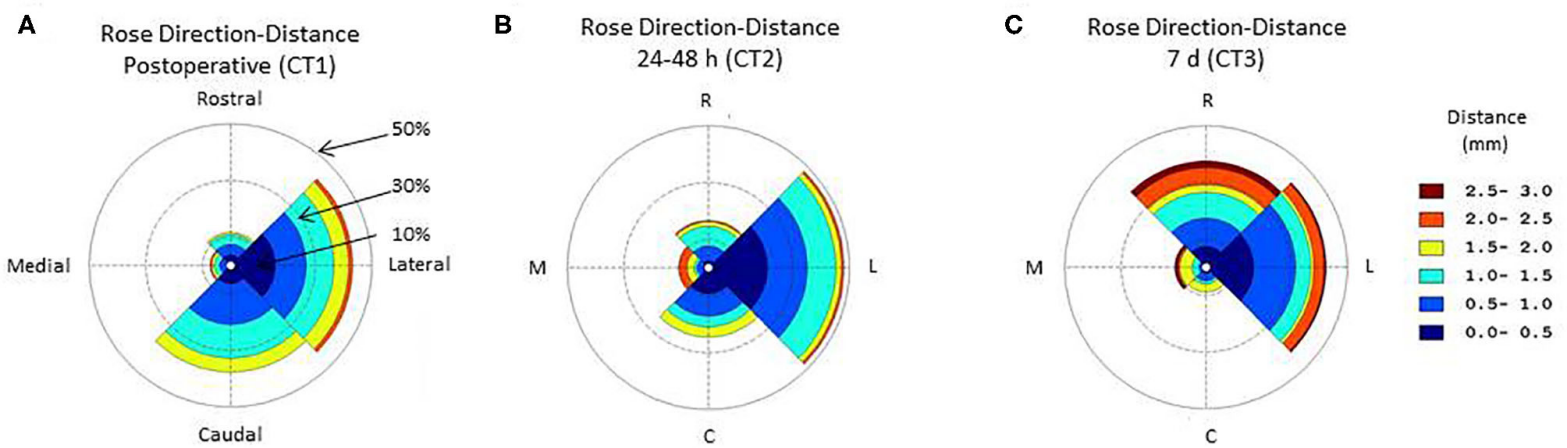
Circular histogram for the orientation and distance of the deep brain stimulation (DBS) electrode lead relative to the theoretical straight path across the sagittal plane. The polar coordinate grid defines 4 quadrants centered toward the rostral, lateral, occipital, medial directions. The distance between the observed and theoretical linear lead paths was grouped (group size, 0.5 mm) and coded according to color. The radial coordinates represent the frequency of a direction and distance deformation. Data are the central tendency over the lead tracks included in the study. **(A)** Immediate post-operative circular histogram for the orientation-distance of the deep brain stimulation (DBS) lead; **(B)** 24–48 h post-operative circular histogram for the orientation-distance of the deep brain stimulation (DBS) lead; **(C)** 7 days post-operative circular histogram for the orientation-distance of the deep brain stimulation (DBS) lead.

The probability of lead deviation varied between the rostral, caudal, medial, and lateral directions in the CT scans immediately (CT1, Friedman, *p* < 0.005) (Figure 1A), 24–48 h (CT2, Friedman, *p* < 0.002) (Figure 1B), and 7 days after lead implantation (CT3, Friedman, *p* < 0.001) (Figure 1C). In the sagittal plane, the immediate post-operative CT scan (CT1) showed higher probability of deviation toward the caudal (0.38) than rostral direction (0.12) (Wilcoxon, *p* < 0.05). The CT scan at 24–48 h (CT2) showed no difference in deviation between the rostral and caudal directions (Wilcoxon, *p* > 0.1). At 7 days (CT3), lead deviation was greater in the rostral (0.37) than caudal direction (0.09) (Wilcoxon, *p* < 0.05). At all 3 times, the coronal CT images showed higher probability of lead deviation in the lateral (0.45) than medial direction (0.11) (Wilcoxon, all times: *p* < 0.005).

**Figure 2:**
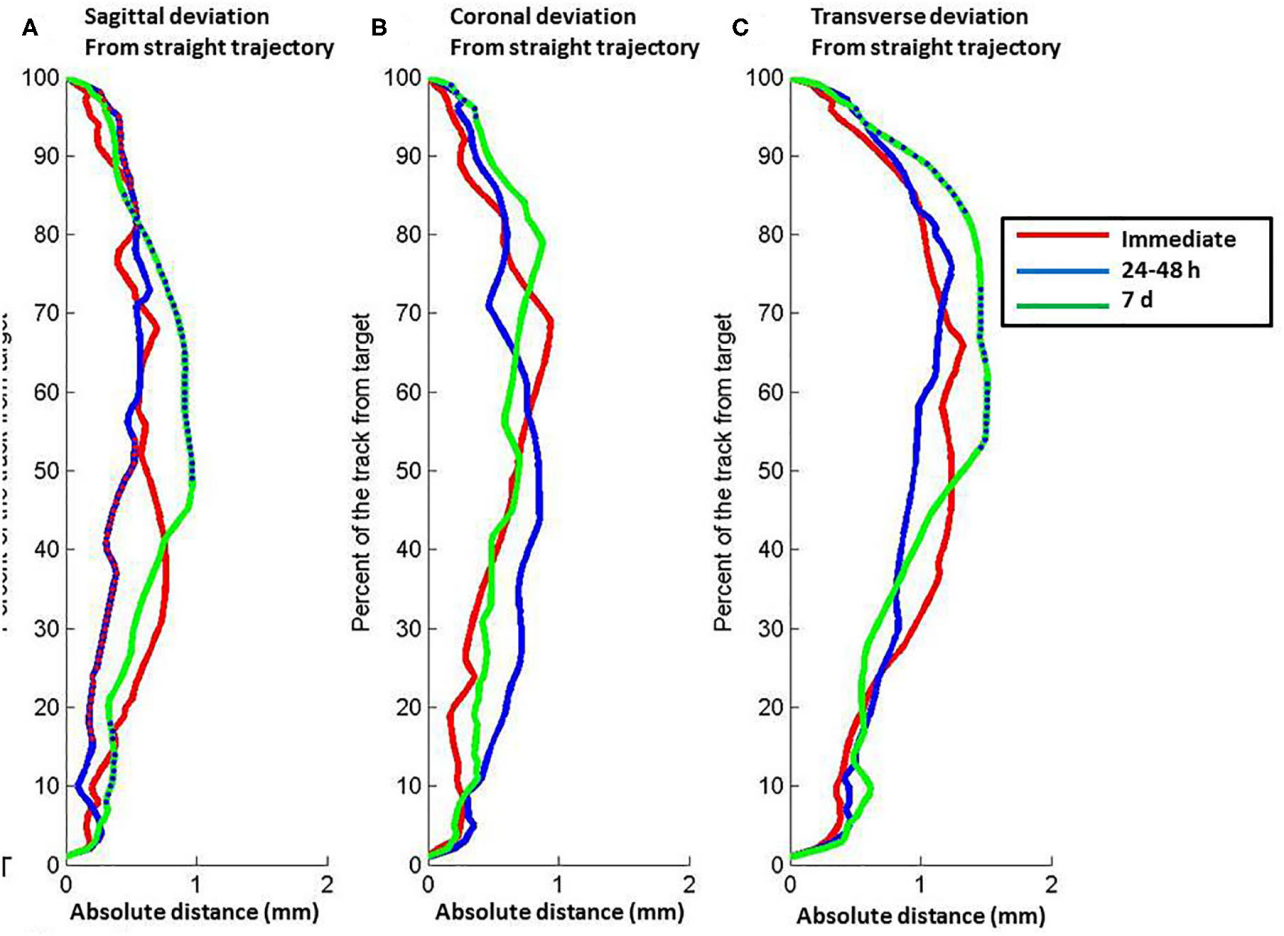
Relation between position along the length of the electrode lead vs. absolute distance of the lead from a theoretical straight path defined by the lead entry site to the target tip. **(A)** Sagittal plane. **(B)** Coronal plane. **(C)** Transverse plane. The lines represent data about lead position from computed tomography scans at different post-operative times: red, immediate post-operative (CT1); blue, 24–48 h post-operative (CT2); green, 7 d post-operative (CT3). Relative depth with significant difference between immediate and 24–48 h post-operative scans are shown by red points on the blue line. Relative depth with significant difference between 24–48 and 7 d post-operative scans are shown by blue points on the green line. Full distance between target and entry point, 100%; target point, 0%.

The lead deviation from the theoretical straight trajectory varied along the lead length from the entry to target point and changed between the successive post-operative CT scans (Freidman, *p* < 0.001) (Figure 2). In the sagittal plane, the half of the lead closest to the target moved toward the straight theoretical trajectory from immediately to 24–48 h (Freidman, *p* < 0.05), the half of the lead closest to the entry point moved away from the straight theoretical trajectory from 24–48 h to 7 days (Freidman, *p* < 0.001) and the quarter of the lead closest to the target moved away from the straight theoretical trajectory from 24–48 h to 7 days after lead implantation (Freidman, *p* < 0.05) (Figure 2A). In the coronal plane, the deviation of the lead from the theoretical straight trajectory changed between successive post-operative CT scans (Friedman, *p* < 0.001); the first 10% of the lead from the entry point moved away from the straight trajectory from 24–48 h to 7 days (Freidman, *p* < 0.05) and only a small part of the most dorsal portion of the lead moved away from the straight theoretical trajectory (Freidman, *p* < 0.05) (Figure 2B). In the transverse plane, the overall position of the lead was significantly different from the straight trajectory (Friedman, *p* < 0.001), and half of the lead closest to the entry point moved away from the straight theoretical trajectory from 24–48 h to 7 days after lead implantation (Freidman, *p* < 0.05) (Figure 2C).

**Figure 3:**
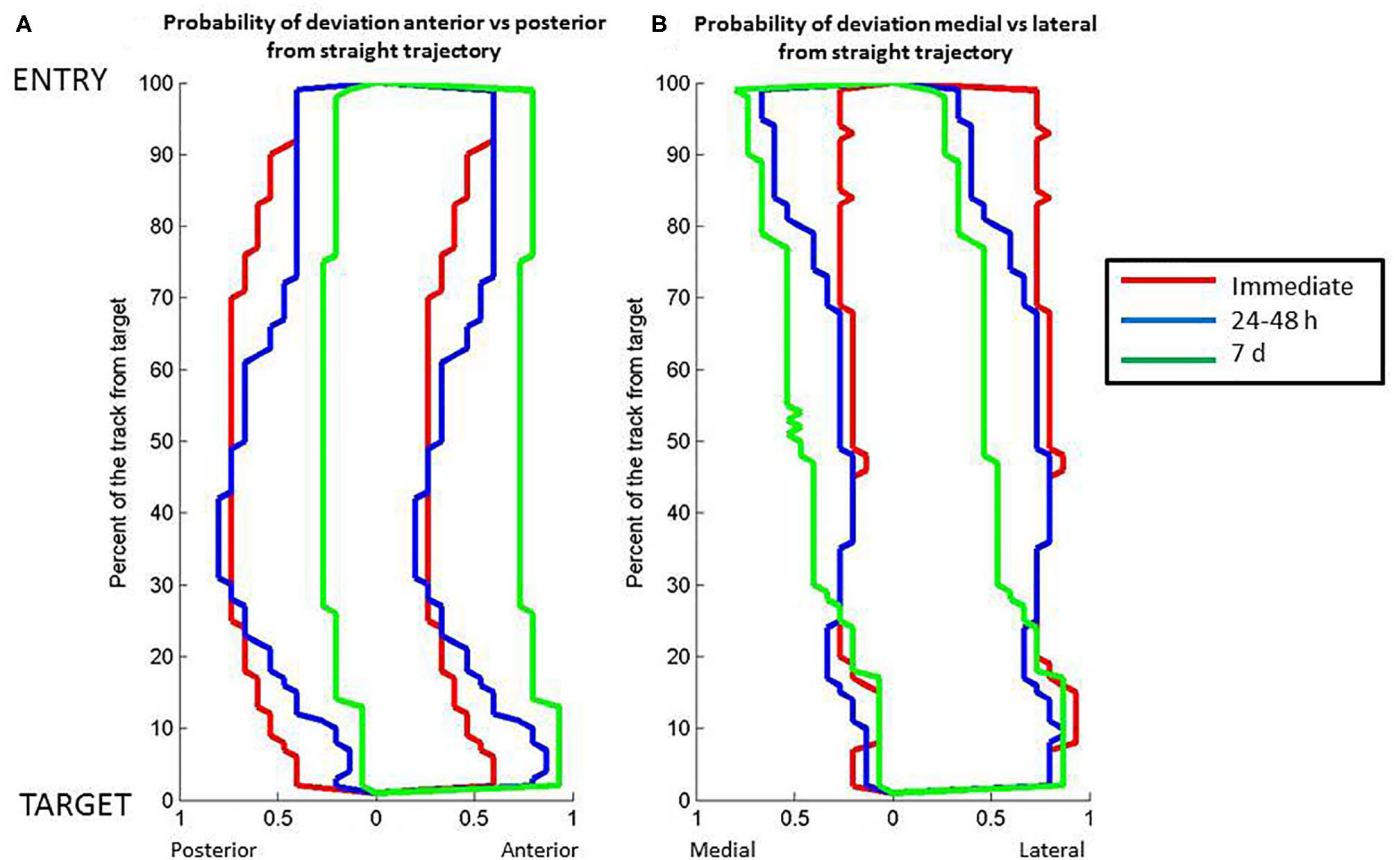
Relation between position along the length of the DBS electrode lead vs. probability of the electrode lead orientation to the theoretical straight path. **(A)** Posterior to anterior orientation. **(B)** Medial to lateral orientation. The lines represent data about lead position from computed tomography scans at different post-operative times: red, immediate post-operative; blue, 24–48 h post-operative; green, 7 days post-operative. Full distance between target and entry point, 100%; target point, 0%.

The overall probability of deviation along the lead from the straight theoretical trajectory varied in the sagittal and coronal planes (Figure 3). In the sagittal plane, the lead deviation in the caudal direction was maximal between 30 and 40% of the lead close to the target with higher probability for the caudal (0.6) than rostral direction (0.4) immediately (Mann-Whitney, *p* < 0.001) and 24–48 h after lead implantation (Mann-Whitney, CT1: *p* < 0.001, CT2: *p* < 0.05). At 7 days, the probability become more homogenous along the lead length but with marked asymmetry, with probabilities of deviation decreased to 0.25 along the entire lead in the posterior direction and >0.75 in the anterior direction (Mann-Whitney, CT3: *p* < 0.001). In the coronal plane, the probability of lead deviation in the caudal direction was homogenous along the lead length, with an asymmetry between the medial (<0.25) and lateral directions immediately after implantation (>0.75) (CT1, Mann-Whitney, *p* < 0.001). The half of the lead close to the entry point moved toward the medial direction (0.5), and there was persistent asymmetry in probability of the half of the lead closest to the target at 24–48 h (CT2, Mann-Whitney, *p* < 0.001) and 7 days after implantation (CT3, Mann-Whitney, *p* < 0.05).

### Subdural Gap

**Figure 4:**
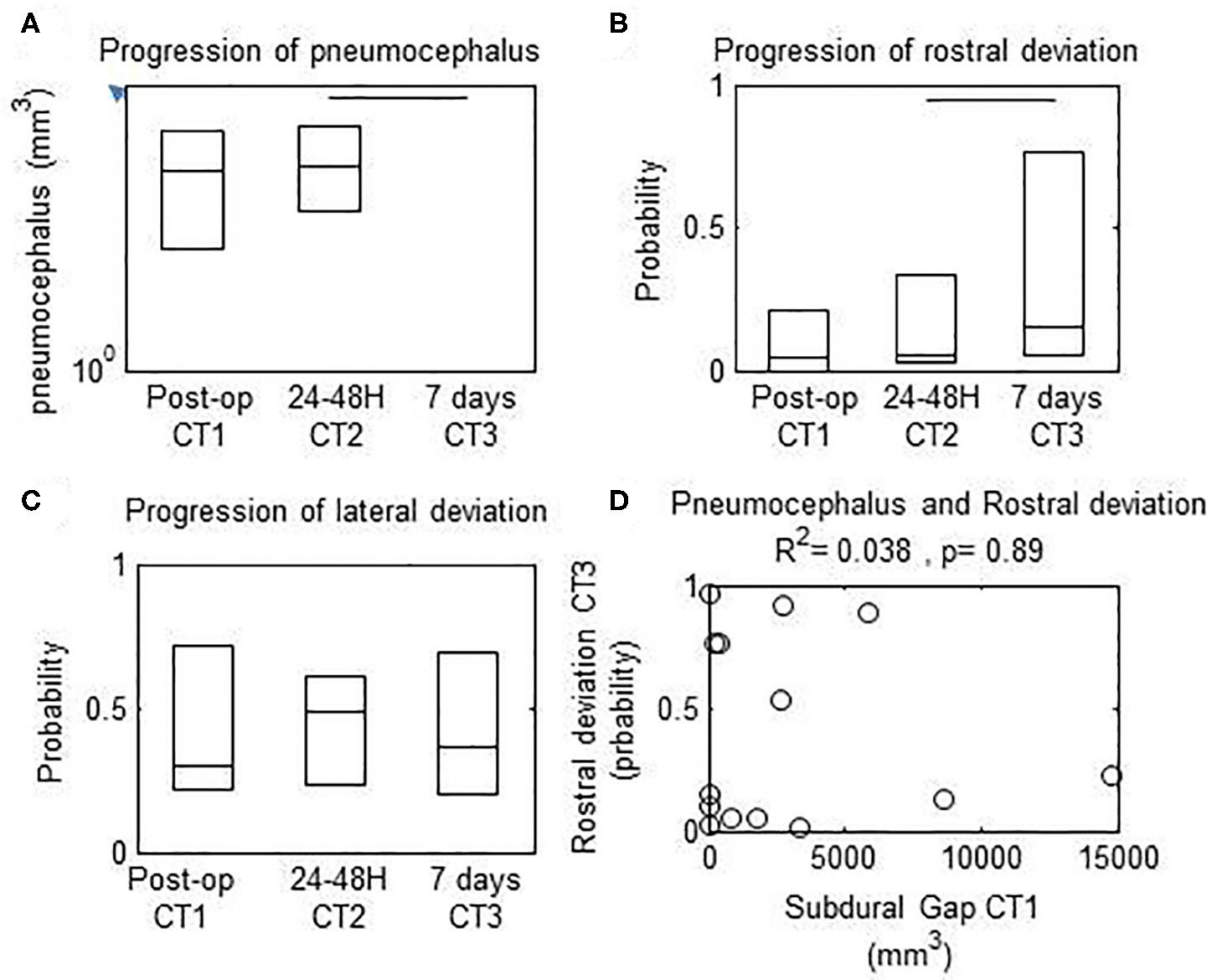
Progression of measured parameters from immediate, 24–48 h, and 7 d post-operative computed tomography (CT) scans. Horizontal bars, significant differences between values at the extremes. **(A)** Subdural gap was decreased between 24–48 h vs. 7 d after surgery. **(B)** Rostral deviation of the lead was increased between 24–48 h vs. 7 d after surgery **(C)** Lateral deviation of the lead was unchanged between 24–48 h vs. 7 d after surgery. **(D)** There was no correlation between probability of rostral deviation of the lead at 7 d vs. pneumocephalus volume on the immediate post-operative CT scan.

The immediate post-operative pneumocephalus was 824.9 mm^3^ (range: 61.4–3202.2 mm^3^) and was unchanged at 24–48 h (median, 338.9 mm^3^ range: 212.6–383.5 mm^3^) (Friedman's test, *p* > 0.1). The pneumocephalus was significantly decreased at 7 days (median, 0 mm^3^; 25th to 75th percentile, 0–0 mm^3^; *p* < 0.001) and resolved in 12 patients (91.7%) (Figure 4). The decrease in pneumocephalus was similar temporally to the increase in rostral (Figure 4B, *Wilcoxon, p* < 0.05) but not lateral deviation of the lead (Figure 4C*, Wilcoxon, p* > 0.1). There was no correlation between the pneumocephalus immediately after implantation and rostral deviation of the lead after 7 days (*Spearman's correlation, p* > 0.1).

### Total Power From Stimulation

All patients had therapeutic benefit from DBS with a decrease of the main motor symptom by at least 2 points on the UPDRS scale. The median power for the ratio of optimal benefit to adverse events was 59.7 μW (range, 43.29–84.17) which is in range with other studies (18). There was no correlation between the total power vs. pneumocephalus volume (Spearman's correlation, *p* > 0.1) or total power vs. maximum deviation (Spearman's correlation, *p* > 0.1).

## Discussion

Our study highlights that the shaft of the DBS electrode is subjected to physical plasticity and that this deformation progresses over the first week. The pattern of this deviation follows some specificities in directionality, time, and depth along the shaft and in a population of patients who benefited from DBS therapy.

The magnitude of lead deformation varied with distance from the entry point. During 24–48 h after surgery, the maximum deviation was located in the middle third of the lead length. The magnitude of the measured deviation was consistent with predictions from theoretical models (19) and results of previous studies (11, 20). At 7 days, the maximum deviation was observed in the first half of the lead length, which overlapped the cortical tissue and corona radiata. Both the sagittal and coronal components of the deviation were increased in the half of the lead adjacent to the entry point (Figure 2).

In the sagittal plan, we distinguished two periods with distinct directionality of the motions (Figure 1). During the initial period after surgery (<48 h), the probability of deviation toward the rostral direction surpasses the probability of deviation toward the caudal direction. This phenomenon was inversed later in the recovery process. One week after surgery, the probability of deviation toward the lateral direction surpasses the probability of deviation toward the medial direction (Figure 2). In the coronal plan, the lateral deviation remained predominant from the immediate post-surgery period up to 1 week later (Figure 1). Over the first 7 days after the surgery, the probabilistic model for the DBS lead localization shows a net anterior movement of the center portion of shaft toward the anterior direction and net movement of the distal part of the shaft toward the medial direction (Figure 3).

All subjects had pneumocephalus after lead implantation. The 2 phases observed in lead deformation were reflected in the evolution of pneumocephalus. The volume of pneumocephalus was constant from immediately to 24–48 h after lead implantation and resolved in most patients by 7 days. The prevalence of deviation rostrally is consistent with the occurrence of brain shift before lead insertion, and the DBS lead shift resulted from resolution of brain shift post-operatively.

The present results support a relation in time between pneumocephalus, brain physical plasticity, and lead deformation (21). The complexity of the mechanisms underlying DBS lead deformation is illustrated by differences in plasticity between cortical and deep nuclei (i.e., mediolateral vs. rostrocaudal) over time. The similar progression of the pneumocephalus and DBS lead shift suggests that loss of cerebrospinal fluid contributes to brain shift and DBS lead deformation (11). In addition, the combined effects of blood flow perturbation, brain tissue swelling along the lead track, and drug effects may contribute to the evolution of mechanical forces on the DBS lead between the entry and target points after implantation (10). Some of these factors such as drugs and loss of cerebrospinal fluid may initiate brain shift before or after the opening of the dura mater. The present results show that the DBS lead bends toward the rostral direction while the pneumocephalus resolves by 7 days after lead implantation (Figure 4). These findings support the possibility that rostral brain shift may occur before lead placement, causing DBS lead deformation while the brain shift resolves. The postulate that the head and brain form a rigid body should be revisited in commercial image-guided neurosurgical systems designated for DBS, as suggested previously (10). As a consequence of perioperative brain shift, a margin is created between the virtual track planed in the preoperative and the track of the DBS lead visualized in post-operative images obtained immediately and 7 days after surgery. Therefore, intraoperative brain shift may be an important limitation of the use of IGNs for accurate DBS lead placement (10, 11). Brain shift also may explain the differences in target localization between image-guided neurosurgical systems and perioperative electrophysiological mapping or microelectrode recording (22, 23) and the discrepancy between imaging-based localization of the DBS lead and clinical outcomes (15). The functional mapping provides an adjustment for target location caused by brain shift that occurs between the dura mater and site of DBS lead placement. Strategies to limit perioperative brain shift and differences between microelectrode recording vs. DBS lead placement may improve the accuracy of DBS lead placement. As previously reported by Morishimoto et al. (24), the surgical technique for placement of the lead fixation device also might contribute to the proximal lead deviation, and technical differences between practitioners may cause different results between studies.

A limitation to our study emerges from the fact that our population of patients met the criteria for normal responder. Therefore, our data provides normative data but does not inform on the prevalence of deviation shift in the non-responder population such as patients with lead relocation. Future studies on non-responders, preferably across multi-centers, is need to elucidate the contribution of brain shift (relative to other factors) as a cause of DBS lead misplacement and poor outcome. Another approach may be to longitudinally investigate, over the years of disease progression, the lead deviation in regard to any changes in DBS therapeutic benefits. At the anatomical level, and in absence of direct visualization of motor area in targets, it will be important to assess the use of diffusion tensor imaging (DTI) to precisely fellow the changes in contact location and relative to anatomic hallmarks (25) rather than in a skull-based coordinate system (23).

In consideration of emerging technologies for intraoperative brain imaging, the present results highlight the need for guidelines to interpret the similarity and differences between the preoperative plan and observed perioperative position of the DBS lead. As all patients received clinical benefit from DBS, the present results suggest that lead deformation does not underlie systemic misplacement of the lead but is a result of brain shifts related to surgical-related events preceding the implantation of the electrode. In absence of intraoperative methods to directly visualize the target or model its shift from the preoperative measurement, it may be justified to combine the use of preoperative imaging, microelectrode recording (MER), and intraoperative imaging to optimize the anatomic and functional accuracy of DBS lead placement. Although the current study provides an estimation of normative lead deviation in a procedure with clinical benefit, multicenter studies and in patient population with larger range of therapeutic benefits are needed to provide clinical guide line on DBS lead deviation. Also, models are needed to be built in respect to the clinical condition and targeted nuclei.

Cortical physical plasticity is important in DBS lead deformation as indicated by the magnitude of deviations observed near the cortical surface. Therefore, perioperative events and anatomic pathology may cause DBS lead deformation. It is unknown whether secondary conditions such as cortical atrophy in patients aged more than 65 years may confound disease severity and limit the benefits from DBS treatment.

## Conclusion

In summary, we documented the normative DBS lead deformation during the first week after implantation. Despite maximum perioperative efforts to prevent brain shift, changes in lead geometry progressed during 7 days after implantation. Further studies documenting lead deformation over time and its relation to anatomic pathology is warranted to improve the post-operative assessment of lead positioning and long-term treatment of patients with DBS. Also, the relevance of lead deformation in patient population with sub-optimal response to DBS therapeutic remains to be established.

## Data Availability Statement

The raw data supporting the conclusions of this article will be made available by the authors under collaborative agreement.

## Ethics Statement

The studies involving human participants were reviewed and approved by IRB University South Alabama. Written informed consent for participation was not required for this study in accordance with the national legislation and the institutional requirements.

## Author Contributions

All authors have collectively contributed to the research design, the data collection, and the interpretation of the data.

## Conflict of Interest

The authors declare that the research was conducted in the absence of any commercial or financial relationships that could be construed as a potential conflict of interest.
